# Waning humoral immunity following monkeypox virus infection and vaccination, Canada, 2020 to 2023

**DOI:** 10.2807/1560-7917.ES.2026.31.14.2500479

**Published:** 2026-04-09

**Authors:** Jérémie Prévost, Sarah J Medina, Ana Citlali Márquez, Kristina Dimitrova, Tahereh Valadbeigy, Gabrielle Angelo P Cortez, Mruthula Narayan, William C Carson, Michael B Townsend, Agatha N Jassem, David Safronetz

**Affiliations:** 1 Special Pathogens Program, National Microbiology Laboratory, Public Health Agency of Canada, Winnipeg, Manitoba, Canada; 2 British Columbia Centre for Disease Control, Vancouver, British Columbia, Canada; 3 Department of Pathology and Laboratory Medicine, University of British Columbia, Vancouver, British Columbia, Canada; 4 Poxvirus and Rabies Branch, Centers for Disease Control and Prevention, Atlanta, United States; 5 Department of Medical Microbiology and Infectious Disease, University of Manitoba, Winnipeg, Manitoba, Canada

**Keywords:** Mpox, Monkeypox virus, MPXV, Orthopoxvirus, Serosurveillance, Serology, Immunity, Antibody, MVA-BN vaccine

## Abstract

**BACKGROUND:**

Monkeypox virus (MPXV) has spread globally to non-endemic countries in recent years and has the potential to cause recurrent outbreaks. Vaccine breakthrough infections and reinfections are suggested to be linked to immunity waning over time.

**AIM:**

We aimed to determine how long individuals remain protected following MPXV infection and if vaccination can be used as a public heath measure to elicit durable protective immune responses.

**METHODS:**

This retrospective observational study investigated the durability of humoral immune responses in a longitudinal cohort of 46 individuals infected with MPXV during the 2022 global mpox outbreak. We collected 86 blood samples up to 7 months after infection, and analysed the antibody responses against MPXV with a serological assay using a panel of eight viral antigens.

**RESULTS:**

Monitoring of antibody kinetics revealed transient IgM responses in the first weeks following infection and a robust polyclonal IgG response that peaked 1–2 months after infection but declined consistently in the following months. Post-exposure immunisation with third-generation modified Vaccinia Ankara-Bavarian Nordic (MVA-BN) vaccine did not seem to increase significantly the strength, breadth or longevity of antibody responses. Using a separate cohort of 25 uninfected long-term MVA-BN vaccinees, we observed low to undetectable seropositivity against most MPXV antigens after 30 months.

**CONCLUSION:**

As circulating antibody titres have been identified as a correlate of protection against mpox, declining antibody levels raise concerns for mpox susceptibility in previously infected and vaccinated persons. This warrants further evaluation of long-term vaccine effectiveness to inform booster vaccination guidance.

**Key public health message**

**What did you want to address in this study and why?**

Monkeypox virus (MPXV) is an emerging pathogen responsible for the mpox disease, which has spread to over 100 countries in 2022, causing more than 130,000 infections It has been suggested that MPXV (re)infections that occur after vaccination or previous MPXV infections have to do with immunity decreasing over time. We therefore wanted to investigate the durability of immune response to MPXV infection and vaccination.

**What have we learnt from this study?**

We developed specific and sensitive assays to track immunity to MPXV infection and vaccination. We showed that individuals infected with MPXV display a strong initial antibody response within the first 2 months after infection, but those antibodies decrease over the following 5 months. A similar decline occurred after mpox vaccination, where the antibodies reached low to undetectable levels after 30 months.

**What are the implications of your findings for public health?**

Decreasing immunity in both MPXV-infected and vaccinated populations suggests that vaccine booster doses may be necessary to maintain antibody levels and protection, in order to reduce the possibility of MPXV reinfections or vaccine breakthrough infections. Future studies need to assess immunity and long-term protection in individuals receiving booster doses of the MVA-BN vaccine compared with the standard two-dose regimen.

## Introduction

Monkeypox virus (MPXV, genus *Orthopoxvirus*, family *Poxviridae*) is an emerging and re-emerging zoonotic viral pathogen responsible for the mpox disease. Mpox is characterised by multiple clinical symptoms including fever, lymphadenopathy and distinctive skin rashes [1]. It presents clinical similarities with the closely related variola virus (VARV) which causes smallpox disease, although mpox generally has milder symptoms and lower case-fatality rates [1]. In recent years, mpox has evolved from a rare zoonosis primarily confined to endemic areas of Central and West Africa to a public health threat of international concern, as outlined by its spread to more than 100 non-endemic countries during the 2022 mpox global outbreak, leading to more than 130,000 reported infections [2]. The resurgence of MPXV can be attributed to various factors, including the cessation of smallpox vaccinations after the disease was considered eradicated in 1980, which led to a decline in immunity against orthopoxviruses (OPXVs) within the general population [3,4].

To this date, smallpox remains the only human disease that has been successfully eradicated using vaccination. Historically, OPXVs less pathogenic for humans, including cowpox virus (CPXV) and vaccinia virus (VACV), have been used as live vaccines against smallpox, given the highly conserved nature of their respective structural proteins [5]. Smallpox vaccines have evolved through three distinct generations, each characterised by differing production methods, safety profiles, and efficacy against VARV and related OPXVs First-generation vaccines were composed of live non-attenuated VACV strains (e.g. NYCBH/Dryvax, Lister/Elstree) grown on animal skin and were used in the worldwide smallpox eradication campaign initiated in the 1960s [5]. Second-generation vaccines (e.g. ACAM2000) were derived from first-generation vaccines in the early 2000s using tissue culture methods [5]. While highly effective, both vaccine generations raised safety concerns which lead to the development of live-attenuated non-replicating third-generation smallpox vaccines, such as modified vaccinia Ankara – Bavarian Nordic (MVA-BN) [5]. MVA-BN is currently approved for immunisation against VARV, MPXV and related OPXV infections in Canada (IMVAMUNE), the United States (US; JYNNEOS) and Europe (IMVANEX) and is recommended by World Health Organization (WHO) for use in at-risk populations, including people living with HIV-1 [6]. As a pre-exposure prophylaxis (PrEP), meta-analyses have estimated the vaccine effectiveness conferred by MVA-BN vaccine at 35% to 86% protection after a single dose and 66% to 90% protection after two doses [7,8]. On the other hand, the use of MVA-BN as a post-exposure prophylaxis (PEP) has not provided protection against infection in most cohorts but did reduce the severity of the symptoms [7,8]. Vaccine boosters are currently not recommended by WHO [9].

It has been suggested that serum antibody titres and neutralising titres are the main correlates of protection against OPXV infections, in both human and non-human primates [10,11]. However, recent reports suggest that the humoral immunity elicited by natural infection with MPXV and current OPXV vaccines tend to decrease over time, which, if humoral immunity is a good predictor of protection, could lead to an increase in susceptibility to MPXV infection and a concomitant decrease in vaccine effectiveness [12-19]. As vaccine breakthrough MPXV infections and reinfections have been recently reported [20-22] we need to better understand antibody kinetics and longevity in the context of MPXV infection and vaccination. Here we report a longitudinal characterisation of the antibody responses elicited in two Canadian cohorts of MPXV-infected and -vaccinated individuals in the context of the 2022 global mpox outbreak.

## Methods

### Serological samples and study participants

Serum samples provided as part of the US Centers for Disease Control and Prevention (CDC) proficiency panel included six samples from de-identified OPXV-naïve individuals, 10 samples from nine MPXV-infected individuals, seven samples from six ACAM2000-vaccinated individuals, and three pools of sera from MVA-BN vaccinees. We used the CDC samples as a proficiency panel, including positive and negative controls, to validate the serological assays. 

The retrospective observational study was designed to use residual sera to assess seroprevalence among individuals with records of MPXV infection and/or OPXV vaccination. Serological samples from MPXV-infected individuals originated from residual sera previously submitted to the British Columbia Centre for Disease Control Public Health Laboratory (BCCDC PHL) for routine clinical testing for syphilis; these samples had previously tested positive for acute MPXV infection by PCR between May 2022 and January 2023 in the Vancouver area. We also obtained serological samples from MVA-BN-vaccinated individuals at the Canadian National Microbiology Laboratory between December 2020 and June 2023 in the Winnipeg area. 

For the infected cohort, no specific criteria such as number of patients (sample size), clinical or demographic characteristics were used for inclusion, beyond PCR-confirmed MPXV infection and being above 18-years-old. Part of the infected cohort also received one or two doses of MVA-BN vaccine as post-exposure immunisation. Vaccinated and unvaccinated subgroups within the MPXV-infected cohort were matched for age, sex and sampling parameters. Details of the statistical analysis are appended in Supplementary Figure S1. For the long-term vaccinated cohort, we collected blood samples from individuals around 5 and 30 months post immunisation with a standard regimen of MVA-BN vaccine. 

Negative control serum samples (naïve) were obtained from anonymised residual sera submitted for routine diagnostic testing for unrelated viral infections; these were from people born after 1980 and without known history of exposure to MPXV or OPXV vaccines.

### Antigens

We selected 11 MPXV antigens to perform serological assessment, listed in Supplementary Table S1. These include MPXV proteins located on the virion external envelope (A35, A36, B6), the internal envelope (A27, A28, A29, E8, H3, M1) and from the viral core (E13, M4). Among these proteins, MPXV A35, B6, A27, A29, E8, H3 and M1 are known targets for OPXV cross-neutralising antibodies [23]. The MPXV A27 and A28 proteins are of particular interest since their VACV orthologues (A25 and A26) are absent from the MVA-BN vaccine genome [24]. The recombinant MPXV proteins used in this study were produced in house or purchased. All protein sequences and numbering are based on the reference sequence MPXV/US/2022/MA001 strain (GenBank accession number: ON563414.3). For membrane-anchored proteins, transmembrane domains were removed to generate a soluble ectodomain version of each protein. We ordered DNA fragments for MPXV A35R, A36R and B6R genes, with the addition of a Twin-Strep tag at the C-terminus, (GenScript, US) and cloned them into the pMT/BiP vector. The MPXV A35, A36 and B6 antigens were produced in insect cells using the Drosophila Expression System (Thermo Fisher Scientific, US) and purified using Strep-tactin XT resin (IBA Lifesciences, Germany). The DNA sequences for MPXV A29L and M4R genes, with the addition of a histidine tag at the N-terminus, were ordered from GenScript and cloned into the pET21a(+) vector. The MPXV A29 and M4 antigens were produced in bacteria using the pET Expression System (Novagen, US) and purified using Ni-NTA Superflow resin (Qiagen, Germany). Recombinant MPXV A28, E8, E13, H3 and M1 were ordered from Biomatik (Canada), with the addition of a N-terminal T7 tag and a C-terminal histidine tag for protein purification. Recombinant MPXV A27 was purchased from Abbexa (United Kingdom).

### Serology assay

For the detection of serum IgG or IgM antibodies against MPXV, we used an indirect ELISA assay. Half area well, high binding flat bottom plates (Corning, US) were coated with recombinant protein at 50 ng per well and incubated overnight at 4 °C. We used bovine serum albumin (BSA) as a negative control protein. Plates were washed with PBS + 0.1% Tween20 (PBST), then incubated with blocking buffer (PBST + 5% skimmed milk) at 37 °C for 1 h. Using blocking buffer as a diluent, serum samples were tested at 1:100, 1:400, 1:1,600 and 1:6,400 dilutions and incubated at 37 °C for 1 h. Plates were washed with PBST, followed by the addition of horseradish peroxidase (HRP)-conjugated goat anti-human IgG or goat anti-human IgM secondary antibodies (SeraCare, US) at a working concentration of 0.5 µg/mL, and incubated for 1 h at 37 °C. After incubation, plates were washed with PBST, and HRP activity was quantified by using the TMB substrate (Thermo Fisher Scientific) before reading the optical density at 650 nm (OD_650_). To determine background and non-specific protein binding, we ran each plate with known positive and negative serum controls. The area under the curve (AUC) was calculated from OD_650_ values obtained with serial serum dilutions, and the signal obtained with BSA was subtracted for each sample. Samples were considered positive when the AUC was greater than the mean AUC plus three standard deviations seen in the negative control wells. This seropositivity threshold accounts for a 99.7% confidence interval for a normal distribution.

### Plaque-reduction neutralisation test

Twofold dilutions of serological samples (1:20 to 1:160) were mixed with 50 plaque-forming units (PFU) of MPXV clade IIb (isolate SP2833) in Dulbecco's Modified Eagle Medium (DMEM) supplemented with 2% fetal bovine serum (FBS). After incubation at 37 °C for 1 h, virus/serum inocula were added to confluent monolayers of Vero E6 cells (ATCC, US) in 12-well plates. All plates were incubated at 37 °C and 5% CO_2_ for 1 h with gentle rocking every 15 min. The inoculum was removed and cells were then overlaid with a solution of minimum essential medium (MEM) supplemented with 2% FBS and 1% CMC. Each condition was tested in duplicate in at least two independent experiments. After 6 days, 100 µL of MTT (3-(4,5-dimethyl-2-thiazolyl)-2,5-diphenyl-2-H-tetrazolium bromide) working solution at a concentration of 5 mg/mL was added to each well and allowed to penetrate the overlay during a 1 h incubation at 37 °C and 5% CO_2_. For each specimen, the average number of plaques was calculated for each dilution and compared with the average number of plaques in infected wells without serum samples. The reciprocal of the highest serum dilution resulting in 50% reduction in plaques compared with controls was defined as the PRNT50 endpoint titre. We considered PRNT50 titres of ≥ 20 positive for MPXV-neutralising antibodies, and titres of < 20 as negative for MPXV-neutralising antibodies.

### Statistical analyses

Data were analysed using GraphPad Prism version 10.4.2 (GraphPad Software, US). We tested every dataset for statistical normality, and used this information to apply the appropriate (parametric or nonparametric) statistical test. Statistical details of experiments are indicated in the figure legends. We considered p values < 0.05 as significant; significance values are indicated as ∗p < 0.05, ∗∗p < 0.01, ∗∗∗p < 0.001, ∗∗∗∗p < 0.0001.

## Results

### Development of a specific and sensitive serological assay to detect monkeypox virus-induced immunity

In order to study the kinetics of the humoral responses elicited against MPXV infection and vaccination, we aimed to develop a sensitive and specific serological assay using recombinant MPXV proteins. Among the 193 open reading frames coded by the MPXV genome, we pre-selected 11 structural proteins which have been identified as immunodominant antibody targets in MPXV-infected or OPXV-vaccinated humans or animals [25,26]. Additional information about the selected MPXV antigens is appended in Supplementary Table S1. 

**Figure 1:**
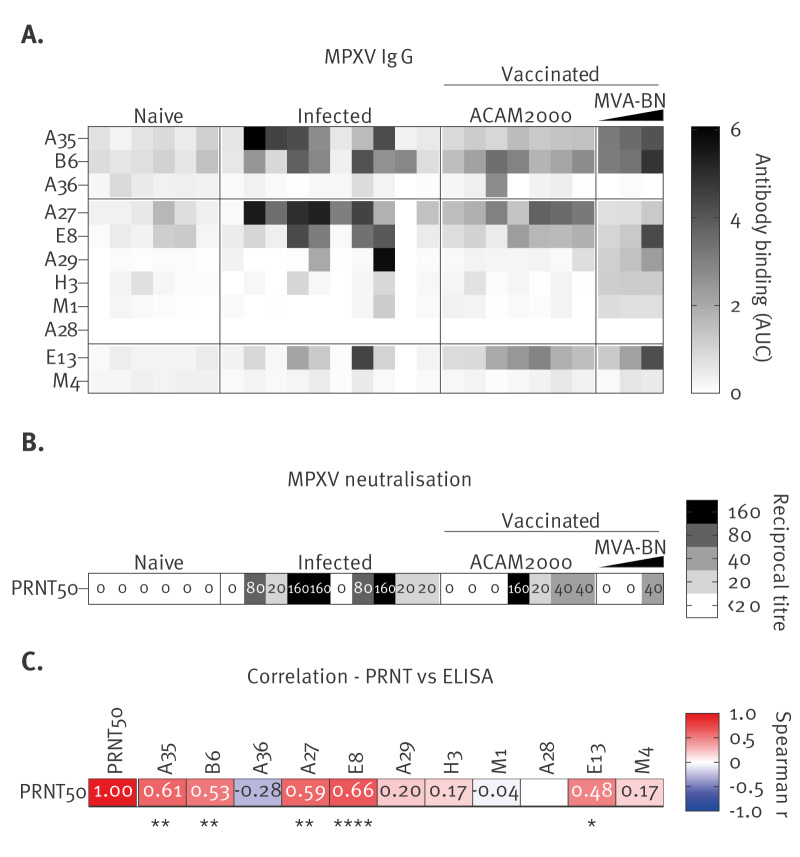
Selection and validation of monkeypox virus antigens for serology assessment, CDC proficiency panel (n = 26 sera)

AUC: area under the curve; CDC: Centers for Disease Control and Prevention; Ig: immunoglobulin; MPXV: monkeypox virus; MVA-BN: modified vaccinia Ankara – Bavarian Nordic; PRNT: plaque reduction neutralisation test.

To begin with, we screened these different MPXV recombinant protein candidates using an indirect ELISA assay against a proficiency panel of OPXV serum samples provided by the US CDC (Figure 1). This serology panel included samples obtained from OPXV-naïve individuals, MPXV-infected individuals, ACAM2000-vaccinated individuals and pooled sera from MVA-BN vaccinees with known levels of OPXV antibodies (Figure 1A). The assay was highly specific since all naïve samples were seronegative against the entire panel of MPXV antigens, while MPXV-infected and vaccinated samples were seropositive for at least one antigen. Eight of the 11 antigens (A35, B6, A27, E8, A29, H3, M1, E13) showed detection of IgG antibody binding in at least one MPXV-infected sample. Since they were not detected by infected sera or pooled MVA-BN-vaccinated sera, the other three antigens (A36, A28, M4) were dropped from further serology testing. The MPXV A27 antigen appeared to show the strongest discrimination between MPXV-infected samples and pooled vaccinated samples, consistent with its absence from the currently used MVA-BN vaccine. On the other hand, MPXV A27 did not discriminate between sera from MPXV-infected individuals and ACAM2000 vaccinees, since this second-generation vaccine strain codes for an intact VACV A25 protein. Interestingly, MPXV A35, B6, E8 and E13 consistently detected IgG antibodies in both infected individuals and vaccinees. 

Panel A: Indirect ELISA was performed using recombinant MPXV proteins (A35, B6, A36, A27, E8, A29, H3, M1, A28, E13, M4), incubated with serological samples from a CDC proficiency panel, including six naïve, 10 MPXV-infected and seven ACAM2000-vaccinated individuals, and three pooled sera from MVA-BN-vaccinated individuals with known levels of OPXV IgG (low, medium, high). MPXV antibody binding was detected using HRP-conjugated anti-human IgG. The specific binding values are shown as AUC, as described in the methods section. Each column represents an individual sample. Panel B: Using a PRNT, the MPXV clade IIb isolate (SP2833) was used to infect Vero E6 cells in presence of serially diluted sera (1:20, 1:40, 1:80, 1:160) from a CDC proficiency panel. The reciprocal of the highest serum dilution resulting in 50% reduction in plaques compared with control wells (without serum) was defined as the PRNT50 endpoint titre. PRNT50 titres ≥ 20 were considered positive for MPXV-neutralising antibodies, whereas titres < 20 were considered negative (0) for MPXV-neutralising antibodies. Panel C: The correlogram summarises pairwise correlations between neutralisation data (PRNT50) and IgG ELISA data (A35, B6, A36, A27, E8, A29, H3, M1, A28, E13, M4) shown in panels A and B. Squares are colour-coded according to the magnitude of the correlation coefficient (r), red squares represent a positive correlation between two variables, and blue squares represent negative correlations. Asterisks indicate all statistically significant correlations (* p < 0.05; ** p < 0.01; **** p < 0.0001). Correlation analysis was done using nonparametric Spearman rank tests.

Furthermore, we performed a seroneutralisation assay (PRNT) using the same proficiency panel for a direct comparison of the sensitivity and specificity of both assays (Figure 1B). We observed strong correlations between neutralising titres and antibody levels detected against MPXV A35, B6, A27, E8 and E13 (Figure 1C), confirming the validity of the ELISA assay. Interestingly, we also noticed that the ELISA assay was more sensitive than the PRNT assay, showing higher seropositivity rates for samples from infected individuals (90% vs 80%), ACAM2000 vaccinees (100% vs 57%) and MVA-BN vaccinees (100% vs 33%). Altogether, we were able to validate the high sensitivity and specificity of our ELISA assay for OPXV serology.

### Humoral responses in monkeypox virus-infected individuals for up to 7 months after infection

To dissect the antibody responses elicited against MPXV infection over time, we used anonymised serological samples obtained from a cohort of confirmed MPXV-infected cases during the 2022 mpox outbreak in Canada. Longitudinal serum samples (n = 86) from 46 individuals presenting typical clinical symptoms of MPXV infection were collected in clinical settings and submitted to BCCDC (Table). Samples were obtained during the acute phase of the infection (n = 12), within 21 days post symptom onset (PSO) as per CDC case definition [27], or from the convalescent phase (n = 74), for up to 224 days PSO (Table). All mpox patients were confirmed to be positive for MPXV by PCR on lesion swab specimens as part of routine laboratory confirmation. All anonymised samples were from adult males, with a median age of 38 years (range: 22–66). A subset of MPXV-infected individuals also received post-exposure vaccination (n = 22) with at least one dose of third-generation MVA-BN vaccine. The majority of them (n = 20) had received the vaccine within 3 weeks before or after symptoms onset.

**Table:** Cohort characteristics of monkeypox virus-infected and MVA-BN-vaccinated individuals, Canada, 2020–2023 (n = 71)

Variable		Infected cohort		Vaccinated cohort (not infected)	
		Not vaccinated	Vaccinated (hybrid immunity)	5 months	30 months
Number of individuals		24	22	5	20
Number of serum samples		46	40	5	20
Median age in years (range)		36 (22–61)	40 (24–66)	30 (25–42)	46 (28–59)
Sex					
Male		24	22	3	11
Female		0	0	2	9
Orthopoxvirus vaccination					
1st generation (Dryvax)		ND	ND	0	3
2nd generation (ACAM2000)		ND	ND	0	2
3rd generation (MVA-BN)	1st dose	0	40	5	19
	2nd dose	0	2	4	17
Median days since last vaccination (range)		NA	110 (11–1,198)	140 (140–140)	886 (886–909)
MPXV PCR positive		46	40	NA	NA
Median days since symptom onset (range)		58 (9–224)	93 (15–185)	NA	NA
Median days between symptom onset and first vaccination (range)		NA	5 (−7 to 43)	NA	NA

MPXV: monkeypox virus; MVA-BN: modified vaccinia Ankara – Bavarian Nordic; NA: not applicable; ND: not disclosed.

**Figure 2:**
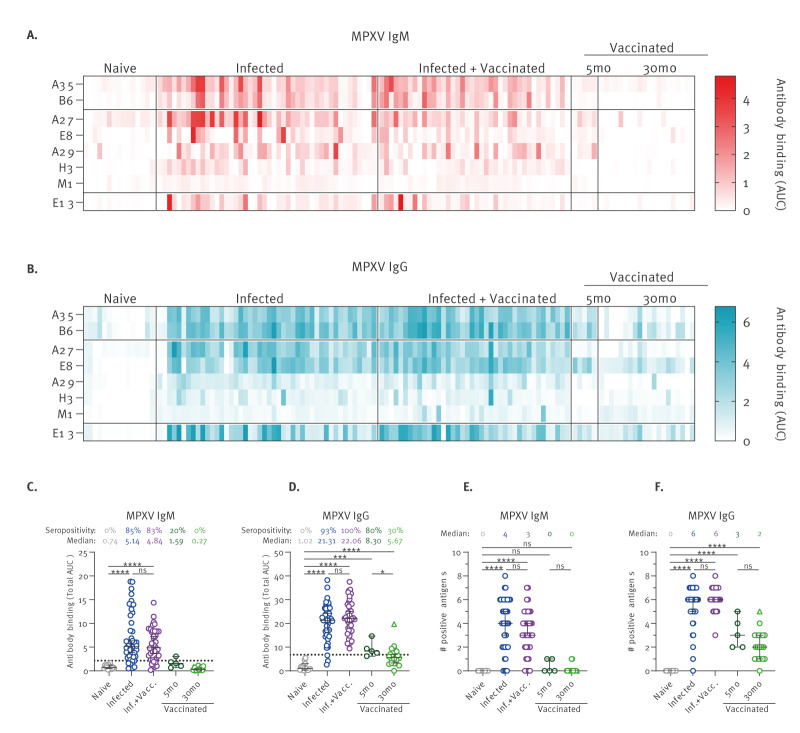
Monkeypox virus infection elicits strong polyclonal antibody responses, Canada, 2020–2023 (n = 86 sera)

AUC: area under the curve; CDC: Centers for Disease Control and Prevention; Ig: immunoglobulin; Inf.+Vacc.: infected and vaccinated; MPXV: monkeypox virus.

We observed that MPXV-infected individuals displayed a strong polyclonal humoral response that was again dominated by IgM and IgG antibodies targeting the A35, B6, A27, E8 and E13 antigens (Figure 2A–B). When combining data for all eight antigens, all infected serum samples were found to be seropositive for MPXV IgM and/or IgG, while OPXV-naïve individuals were all seronegative (Figure 2C–D). Among the 86 infected sera, three samples from acutely infected individuals were ruled IgM+/IgG−, while the rest of the cohort were either IgM+/IgG+ (n = 69) or IgM−/IgG+ (n = 14). Infected samples showed IgM seroreactivity against a median of four antigens and IgG seroreactivity against a median of six antigens (Figure 2E–F). Curiously, post-exposure administration of the MVA-BN vaccine did not seem to significantly affect the total antibody levels or the breadth of the antibody response against MPXV infection (Figure 2C–F).

Indirect ELISA was performed using recombinant MPXV proteins (A35, B6, A27, E8, A29, H3, M1, E13), incubated with 15 naïve sera, 46 MPXV-infected sera, 40 MPXV-infected and vaccinated (Inf. + Vacc.) sera, and 25 vaccinated sera collected at 5 months (5mo) or 30 months (30mo) post vaccination. MPXV antibody binding was detected using HRP-conjugated (A) anti-human IgM or (B) anti-human IgG. The specific binding values are shown as AUC. Each column represents an individual sample. For each sample, (C, D) the magnitude and (E, F) the breadth of the (C, E) IgM and (D, F) IgG antibody responses were calculated based on (C, D) the cumulative AUC for all eight MPXV proteins tested and (E, F) the number of antigens for which the AUC was above the seropositivity threshold. Error bars indicate median values ± 95% confidence intervals. The dotted lines represent seropositivity thresholds. Statistical significance was tested using Mann–Whitney U tests (∗ p < 0.05; ∗∗∗ p < 0.001; ∗∗∗∗ p < 0.0001; ns: non-significant).

**Figure 3:**
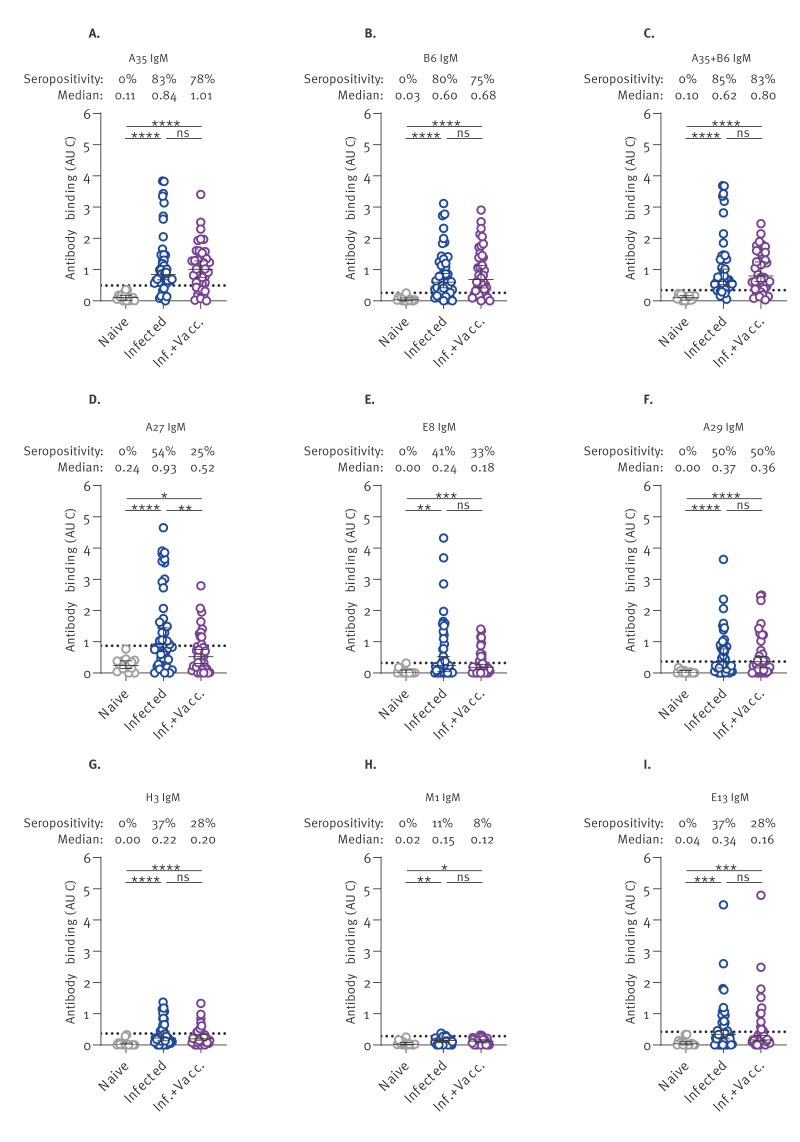
IgM responses in monkeypox virus-infected individuals, Canada, 2022–2023 (n = 46 sera)

When focusing specifically on the IgM responses (Figure 3), we noticed that antibodies against the A35 and B6 antigens were the most prevalent among MPXV-infected individuals, exhibiting at least 80% seropositivity and reaching 85% seropositivity when combined (Figure 3A-C). Interestingly, these two glycoproteins are the only ones from our panel that are localised on the outer membrane of the virus, possibly making them more accessible to the immune system. The localisation of MPXV proteins is listed in Supplementary Table S1. Detection of IgM antibodies against the other six antigens (A27, E8, A29, H3, M1, E13) was less consistent, with seropositivity rates ranging from 11% to 54% (Figure 3D–I). Individuals who were both infected and vaccinated showed comparable IgM responses against all antigens tested, except for A27 IgM, which were found in a larger proportion of infected individuals (Figure 3A–I). This could be due to the absence of the VACV A25 protein in the MVA-BN vaccine, leading to the redirection of the immune responses against other OPXV antigens. 

Ig: immunoglobulin; Inf.+Vacc.: infected and vaccinated; MPXV: monkeypox virus.

Indirect ELISA was performed using recombinant MPXV proteins (A) A35, (B) B6, (C) A35 and B6 (D) A27, (E) E8, (F) A29, (G) H3, (H) M1 and (I) E13, incubated with 15 naïve sera, 46 MPXV-infected sera and 40 MPXV-infected and vaccinated (Inf. + Vacc.) sera. MPXV antibody binding was detected using HRP-conjugated anti-human IgM. The specific binding values are shown as areas under the curve. Error bars indicate median values ± 95% confidence intervals. The dotted lines represent seropositivity thresholds. Statistical significance was tested using Mann–Whitney U tests (∗, p < 0.05; ∗∗, p < 0.01; ∗∗∗, p < 0.001; ∗∗∗∗, p < 0.0001; ns, non-significant).

**Figure 4:**
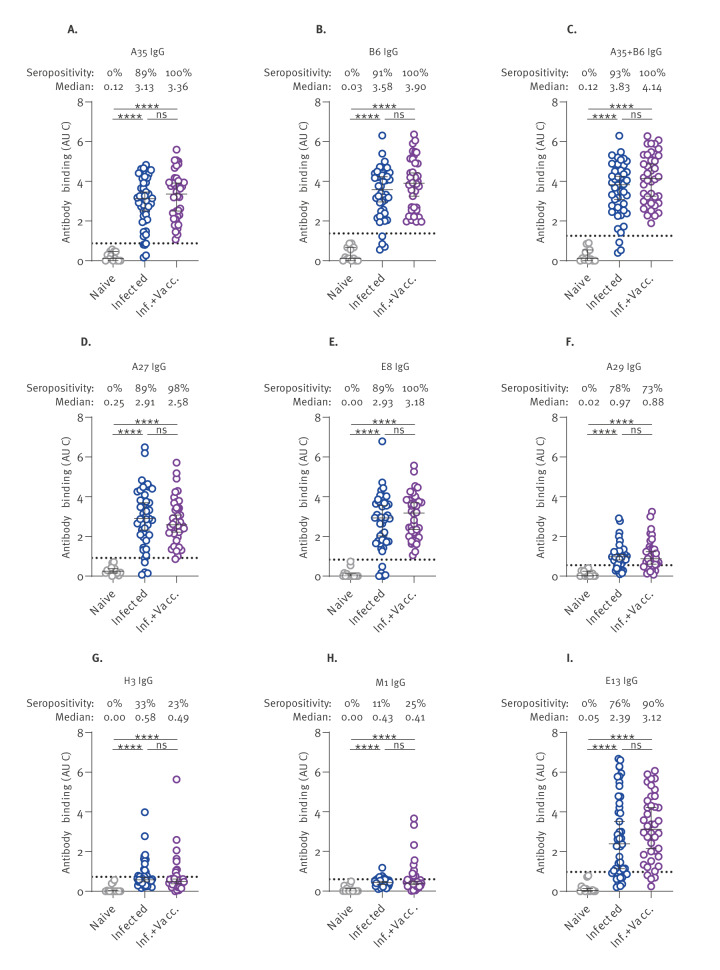
IgG responses in monkeypox virus-infected individuals, Canada, 2022–2023 (n = 46 sera)

When focusing specifically on the IgG responses (Figure 4), we observed a broader polyclonal response, with six antigens (A35, B6, A27, E8, A29, E13) reaching at least 75% seropositivity among MPXV-infected individuals, although antibodies levels were far superior for five of them (A35, B6, A27, E8, E13), as compared with A29 IgG (Figure 4A–I). In line with IgM, the use of MVA-BN post-exposure vaccination did not significantly increase the levels of IgG against any antigen (Figure 4A–I). In the end, we successfully identified five immunodominant antigens (A35, B6, A27, E8 and E13) targeted by the humoral response during natural MPXV infection.

Ig: immunoglobulin; Inf.+Vacc.: infected and vaccinated; MPXV: monkeypox virus.

Indirect ELISA was performed using recombinant MPXV proteins (A) A35, (B) B6, (C) A35 and B6 (D) A27, (E) E8, (F) A29, (G) H3, (H) M1 and (I) E13, incubated with 15 naïve sera, 46 MPXV-infected sera and 40 MPXV-infected and vaccinated (Inf. + Vacc.) sera. MPXV antibody binding was detected using HRP-conjugated anti-human IgG. The specific binding values are shown as areas under the curve. Error bars indicate median values ± 95% confidence intervals. The dotted lines represent seropositivity thresholds. Statistical significance was tested using Mann–Whitney U tests (∗∗∗∗ p < 0.0001; ns: non-significant).

Afterwards, we studied how MPXV IgM and IgG antibody levels fluctuate over time, with an emphasis on the immunodominant responses. Since the antibody responses were only marginally affected by MVA-BN post-exposure vaccination (Figures 3 and 4), we included all 86 samples from infected individuals in the analyses. Following a typical antibody isotype switching, IgM+/IgG− samples were collected at a median of 10 days PSO (range: 9–12), IgM+/IgG+ at a median of 71 days PSO (interquartile range (IQR): 34–114), and IgM−/IgG+ at a median of 166 days PSO (IQR: 116–175). 

**Figure 5:**
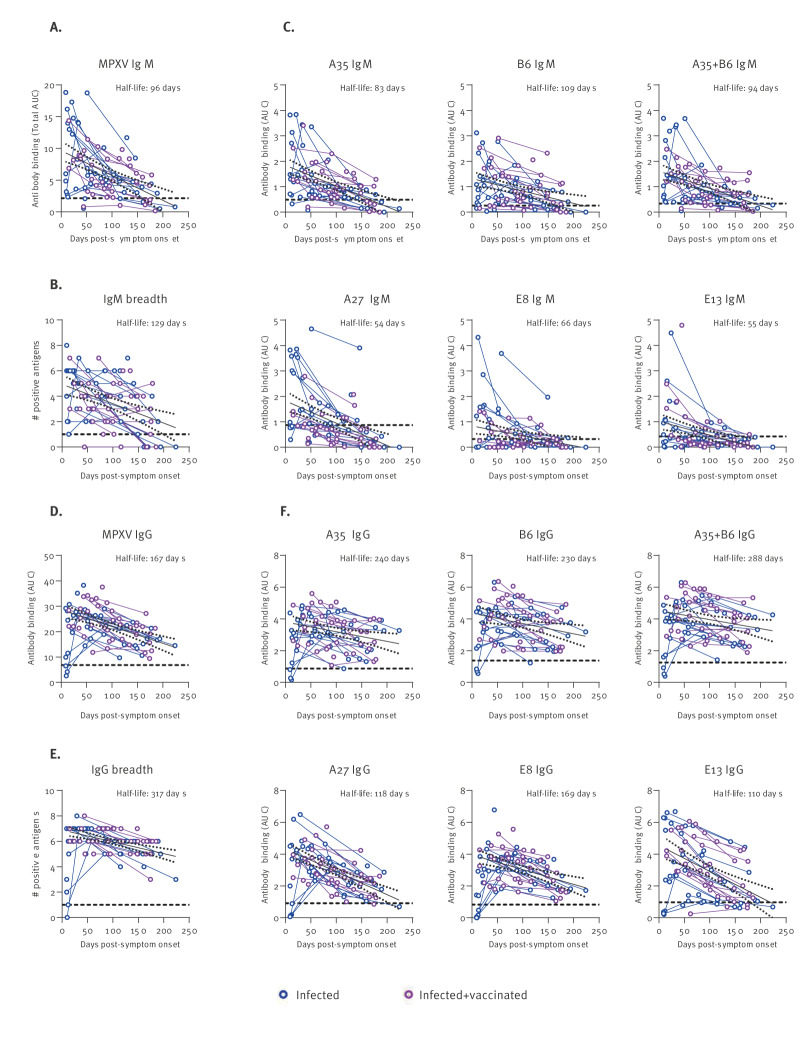
The magnitude and breadth of monkeypox virus-specific antibody responses decrease over time, Canada, 2020–2023 (n = 86 sera)

AUC: area under the curve; Ig: immunoglobulin; MPXV: monkeypox virus.

The overall IgM response against MPXV peaks in the first 2–4 weeks PSO, followed by a gradual decline with a half-life of 96 days, reaching low to undetectable levels after 5–6 months PSO (Figure 5A). The breadth of the IgM response followed a similar downward trend (Figure 5B). When looking at the antibody kinetics against individual antigens, we noted that A35 and B6 IgM had a prolonged half-life (94 days) as compared with A27, E8 and E13 IgM (54–66 days) (Figure 5C). 

Indirect ELISA was performed using recombinant MPXV proteins (A35, B6, A27, E8, A29, H3, M1, E13) incubated with 46 MPXV-infected sera and 40 MPXV-infected and vaccinated sera. MPXV antibody binding was detected using HRP-conjugated (A–C) anti-human IgM or (D–F) anti-human IgG. The specific binding values are shown as AUC. (A, D) The overall magnitude of the antibody responses was calculated based on the cumulative AUC for all eight MPXV proteins tested. (B, E) For each sample, the breadth of the antibody responses was calculated based on the number of antigens for which the AUC was above the seropositivity threshold. (C, F) Graphs represent the antibody responses against selected antigens (A35, B6, A35 and B6, A27, E8, E13). For all graphs, AUC values are plotted against the number of days between symptoms onset and blood collection. Values from paired samples are connected with blue or purple lines. Antibody decay modelling was performed using linear regression for optimal curve fitting. Linear regression is depicted as solid black lines and 95% confidence intervals as dotted black lines. Antibody half-life values were inferred using the plotted linear function. The horizontal dashed lines represent seropositivity thresholds. (D–F) Serum samples collected within 2 weeks post symptom onset were excluded from linear regression analyses. Paired samples from the same individual are shown as connected dots.

In contrast, the IgG responses elicited upon MPXV infection were poorly detected in the first 2 weeks PSO, peaking later around 4–8 weeks PSO (Figure 5D). The MPXV IgG also persisted longer than MPXV IgM, with a half-life of 167 days, and were still detectable in samples collected up to 7 months PSO, although the antibody levels were roughly two times lower. At their maximum, infected samples were seroreactive against all eight antigens tested, slowly losing the polyclonal breadth afterwards (Figure 5E). Strikingly, we observed that A35 and B6 IgG had a half-life of 288 days, more than twice the longevity of A27 IgG (118 days) and E13 IgG (110 days), and substantially superior to E8 IgG (169 days) (Figure 5F). Altogether, our results suggest that humoral immunity against MPXV infection is waning over time.

### Humoral responses in MVA-BN vaccinees during long-term follow-up

**Figure 6:**
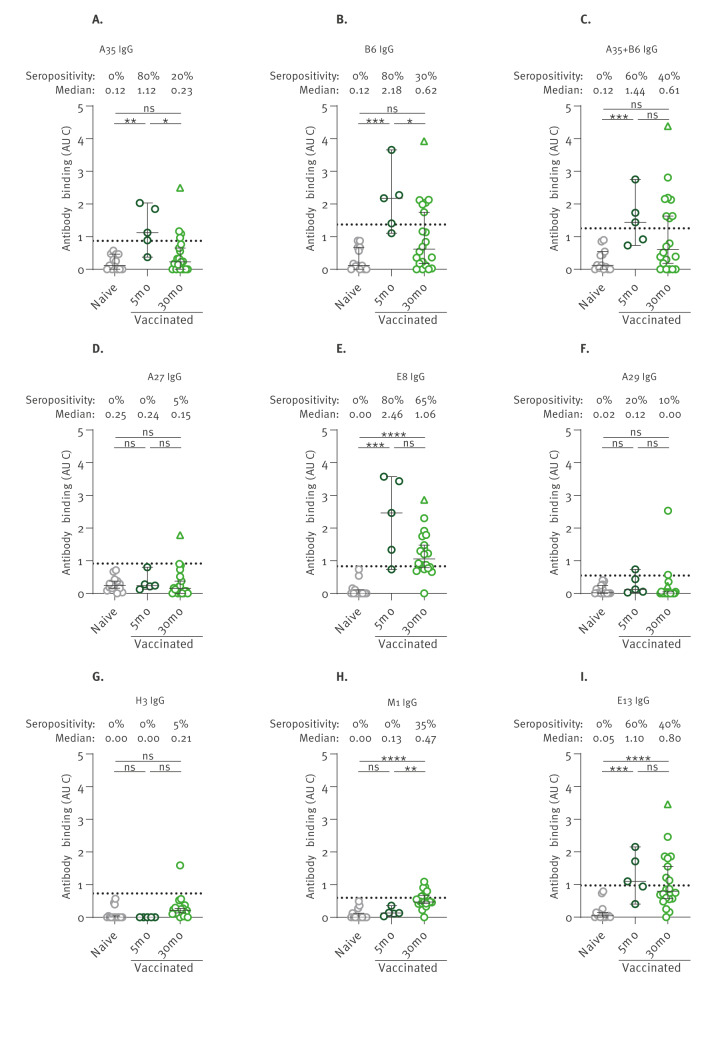
Seropositivity rate in long-term MVA-BN vaccinees, Canada, 2020–2023 (n = 25 sera)

AUC: area under the curve; Ig: immunoglobulin; MPXV: monkeypox virus.

To get a better understanding of the longevity of the cross-reactive antibody responses elicited by OPXV vaccination, we used serological samples obtained from a small cross-sectional cohort of OPXV vaccinees with risk of occupational exposure to MPXV (Table). Most vaccinated individuals received a standard regimen of MVA-BN vaccine, except for participants who had a history of vaccination with first- or second-generation OPXV vaccines, who received a single dose of MVA-BN. Serum samples were collected around 5 months (n = 5) or 30 months (n = 20) after immunisation and were assessed for MPXV serology. Since these are long-term follow-up samples, the detection of MPXV IgM showed only 20% seropositivity at 5 months and 0% at 30 months after immunisation (Figure 2C). This is in agreement with the poor IgM levels in MPXV-infected people after 5 months PSO (Figure 5A). On the other hand, 80% of the 5 months vaccinees were seropositive for MPXV IgG against a median of three antigens, as compared with 30% seropositivity for 30 months vaccinees against a median of two antigens (Figure 2D,F). Similarly to MPXV-infected individuals, the IgG responses elicited by vaccination was also dominated by A35, B6, E8 and E13 antibodies, but not A27, since MVA-BN lacks its expression (Figure 6A–I). The levels of antibodies against these immunodominant antigens all decreased between 5 and 30 months post immunisation. At 30 months, the highest seropositivity rate and antibody levels were observed against the E8 antigen (Figure 6E). Of note, one participant who had previously received one dose of ACAM2000 and one dose of MVA-BN (depicted as a triangle symbol) was shown to have far superior MPXV IgG responses compared with the other 30 months vaccinees, including seroreactivity to the A27 antigen (Figures 2C–F, Figure 6). This is consistent with the antibody responses observed with other ACAM2000 vaccinees (Figure 1A). In brief, the cross-reactive antibody responses elicited by OPXV vaccination decrease over time in a similar fashion as MPXV-induced immunity.

Indirect ELISA was performed using recombinant MPXV proteins (A) A35, (B) B6, (C) A35 and B6 (D) A27, (E) E8, (F) A29, (G) H3, (H) M1 and (I) E13, incubated with 15 naïve sera and 25 vaccinated sera collected at 5 months (5mo) or 30 months (30mo) post vaccination. MPXV antibody binding was detected using HRP-conjugated anti-human IgG. The specific binding values are shown as AUC. Error bars indicate median values ± 95% confidence intervals. The dotted lines represent seropositivity thresholds. Statistical significance was tested using Mann–Whitney U tests (∗ p < 0.05; ∗∗ p < 0.01; ∗∗∗ p < 0.001; ∗∗∗∗ p < 0.0001; ns: non-significant).

## Discussion

Gaining a better understanding of the kinetics and durability of the immune responses directed against MPXV, elicited either by natural infection or by vaccination, and the protection afforded by these antibodies, is critical for the effective management of active outbreaks and the prevention of future large-scale outbreaks. The current observational study sheds light on the decay of MPXV humoral immunity in confirmed mpox cases and MVA-BN vaccinees, several months after infection or vaccination. Here we show that serum antibodies from a cohort of 46 MPXV-infected individuals are waning at a constant rate during the convalescent phase of the infection, despite an early robust polyclonal antibody response. More importantly, we observed MPXV antibody waning in longitudinal samples from 28 infected individuals with or without MVA-BN post-exposure vaccination. These findings are supported by another group which observed the decline of neutralising antibodies between 1 month and 8 months post infection in paired sera from four mpox convalescent individuals [18]. Interestingly, our results also suggest that post-exposure vaccination with MVA-BN may not improve humoral immunity in MPXV-infected individuals. Long-term follow-up studies with larger cohorts are needed to confirm if MPXV humoral immunity keeps waning beyond 7 months post infection, and if this is linked to an increase in the risk of reinfection. As at March 2026, only few cases of MPXV reinfection have been reported, but they could be increasing over time given the concomitant decrease in humoral immunity [22]. An OPXV vaccination may be required in MPXV-experienced individuals if declining serum antibodies result in reduced protection.

In contrast with infection-induced immunity, the longevity of the antibody responses following MVA-BN vaccination has been extensively characterised, with multiple groups reporting the diminution of antibody titres within the first 12 months after vaccination [12-19]. In this study, we observed that MPXV-specific antibodies elicited by the MVA-BN vaccine keep decreasing for up to 2.5 years after vaccination. The durability of the protection afforded by OPXV vaccination has been linked to antibody titres, and it has been estimated that the vaccine effectiveness of a two-dose MVA-BN regimen will drop by 10% within the first 2 years after immunisation [10]. Losses in protection could be restored by providing a booster dose of MVA-BN at the 2-year mark [10]. A report in 2024 has noted limited vaccine breakthrough infections among fully vaccinated individuals (< 1%) [20], and by March 2026, there have been no booster vaccination recommendations by WHO and other public health organisations, except for laboratory workers at high risk of occupational exposure 2 years after a primary series [28]. Should monitoring detect increasing rates of breakthrough infections that correlate with antibody losses as observed here, vaccine recommendations may need to be revised to include MVA-BN booster doses. Next-generation multivalent mRNA vaccines against MPXV are currently being developed [29,30], and have entered Phase I/II clinical trials (NCT05988203, NCT05995275) to assess their safety and immunogenicity. It would be interesting to compare the durability of the immune responses elicited by this vaccine platform compared with currently used live-attenuated vaccines.

As previously reported, none of the available commercial serological assays are able to distinguish mpox-positive from vaccinated samples [31]. Here we successfully developed highly specific and sensitive in-house serological assays using recombinant MPXV proteins. These assays are expected to be uniformly effective across all MPXV clades (Ia, Ib, IIa and IIb) given the high degree of conservation (> 98%) of the selected antigens. Importantly, our results suggest the use of MPXV A27 for future serosurveillance studies since it acts as an immunodominant antigen able to discriminate the antibody responses elicited during infection and vaccination. Indeed, we observed a seropositivity rate of 93% in the entire cohort of MPXV-infected individuals, while none of the MVA-BN vaccinees showed seroreactivity against this antigen. However, this was specific for participants who received the MVA-BN vaccine exclusively, while the majority of ACAM2000 vaccinees had detectable IgG antibodies against MPXV A27. People who were immunised with first-generation vaccines have also been shown to be seroreactive for its VACV orthologue [25]. Of note, previous records have also identified discriminating antigens, including MPXV A27 and B21 as serological markers for MPXV infection, and MPXV M1 for MVA-BN vaccination [26,32]. Interestingly, a third-generation vaccine currently approved against mpox and smallpox in Japan (LC16m8) and recommended by WHO [6] lacks the expression of another immunodominant antigen (VACV B5) [33]. Given that we have detected antibodies against MPXV B6 in 96% of MPXV-infected participants, it could probably be used as a discriminating antigen in the context of LC16m8 vaccination. Finally, to assess long-lasting immunity, MPXV A35 and B6 seem to be preferable in the context of infection, due to their extended half-life, while MPXV E8 would be preferable in the context of MVA-BN vaccination, due to its higher seroreactivity rate at 30 months post vaccination.

A limitation of the current study is that the cohort of mpox-positive individuals is composed exclusively of adult males. Therefore, it would be valuable to investigate the MPXV-induced immunity in females, but also in children, since this age group has the highest MPXV incidence in endemic regions [2]. Another limitation is that we only looked at the waning of circulating antibodies, while antigen-specific memory B and T-cells could also be good indicator of long-lasting immunity [17,34]. However, we did not have access to whole blood samples to perform such analyses. Further studies are required to assess the longevity of cellular immunity and its role in protection against MPXV infection. In the end, we acknowledge that some confounding factors could have influenced the observed antibody responses, including mpox disease severity, HIV-1 status, or intravenous vaccinia immunoglobulin (VIG-IV) treatment. Unfortunately, these parameters were not disclosed for anonymised samples.

## Conclusion

This study provides a characterisation of the magnitude and durability of MPXV-specific humoral immunity following natural infection and MVA-BN vaccination. Our findings show that both infection- and vaccine-induced antibodies decline over time. This raises important considerations for long-term vaccine protection, the potential need for booster immunisations, and the risk of reinfection. Moreover, the development and validation of highly specific MPXV serological assays using discriminating antigens offer valuable tools for future serosurveillance studies.

## Data Availability

The data that support this study’s findings are available from the corresponding authors upon reasonable request.

## Supplementary Data

165000 Supplement
