# Supplementary material for: Waning humoral immunity following monkeypox virus infection and vaccination, Canada, 2020 to 2023
Source: Euro Surveill. 2026 Apr 9;31(14):2500479. doi: 10.2807/1560-7917.ES.2026.31.14.2500479 (PMC13074483; doi:10.2807/1560-7917.ES.2026.31.14.2500479)
Supplement: Supplement [file 25-00479_PREVOST_Supplement.pdf]

This supplementary material is hosted by *Eurosurveillance* as supporting information alongside the article "**Waning Humoral Immunity Following Monkeypox Virus Infection and Vaccination**" on behalf of the authors, who remain responsible for the accuracy and appropriateness of the content. The same standards for ethics, copyright, attributions and permissions as for the article apply. Supplements are not edited by *Eurosurveillance* and the journal is not responsible for the maintenance of any links or email addresses provided therein.

Table S1. Selected MPXV antigens for serological assays

MPXV antigen	Coding gene	VACV ortholog	Viral particle localization	Function	Expression system	Recombinant protein residues	% similarity with MVA-BN vaccine*	% conservation between MPXV clades#
A35	A35R	A33	Outer membrane	C-type lectin	Insect cells (Drosophila S2)	Arg58-Thr181	92.4 %	98.3 %
A36	A36R	A34	Outer membrane	C-type lectin	Insect cells (Drosophila S2)	Tyr38-Lys168	96.4 %	100 %
B6	B6R	B5	Outer membrane	Viral release	Insect cells (Drosophila S2)	Thr20-Glu275	96.2 %	100 %
A27	A27L	A25	Inner membrane	Fusion suppressor	Bacteria (E.coli)	Leu428-Thr695	Absent	99.1 %
A28	A28L	A26	Inner membrane	Fusion suppressor	Bacteria (E.coli)	Met1-Asp406	Absent	98.6 %
A29	A29L	A27	Inner membrane	Attachment	Bacteria (E.coli)	Ser21-Asn84 (Cys71Ala, Cys72Ala)	93.6 %	98.2 %
E8	E8L	D8	Inner membrane	Attachment	Bacteria (E.coli)	Met1-Ala261	94.7 %	99.0 %
H3	H3L	H3	Inner membrane	Attachment	Bacteria (E.coli)	Met1-Phe282	94.8 %	99.1 %
M1	M1R	L1	Inner membrane	Fusion	Bacteria (E.coli)	Met1-Glu185	98.8 %	100 %
E13	E13L	D13	Virion core	Capsid	Bacteria (E.coli)	Met1-Asn551 (Asp513Gly)	98.9 %	99.1 %
M4	M4R	L4	Virion core	Nucleocapsid	Bacteria (E.coli)	Met1-Asp251	98.4 %	99.6 %

MPXV, Monkeypox virus; MVA-BN, Modified Vaccinia Ankara – Bavarian Nordic; VACV, Vaccinia virus
*Based on the alignment of protein sequences from the MPXV/USA/2022/MA001 reference strain (Genbank accession number: ON563414.3) and the MVA-BN vaccine strain (Genbank accession number: DQ983238.1).
#Based on the alignment of protein sequences from MPXV clade Ia, Ib, IIa and IIb reference strains (Genbank accession number: DQ011155.1, PQ762586.1, DQ011157.1, ON563414.3)

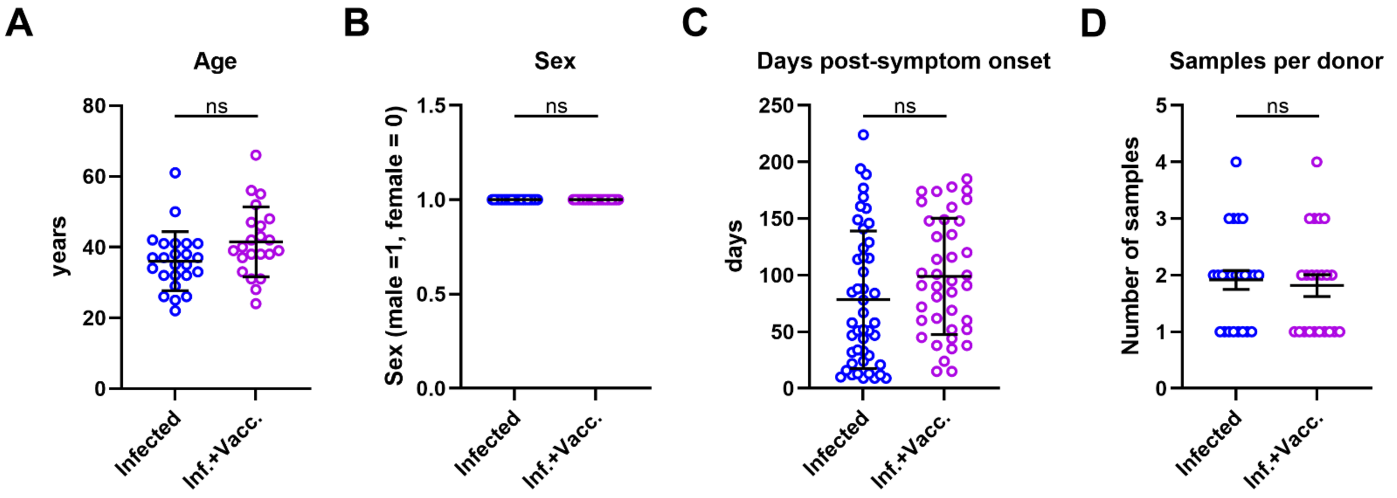

Figure S1. MPXV-infected cohorts were matched for age, sex and sampling.
Groups of MPXV-infected (n=24) and MPXV-infected and vaccinated (Inf.+Vacc., n=22) individuals were matched for (**A**) age, (**B**) sex, and (**C-D**) sampling parameters. Error bars indicate mean values ± standard deviation (SD). Statistical significance was tested using a Mann-Whitney U test or a t test based on statistical normality (ns, non-significant).
